# A New Wearable Device for Blood Pressure Estimation Using Photoplethysmogram

**DOI:** 10.3390/s19112557

**Published:** 2019-06-04

**Authors:** Remo Lazazzera, Yassir Belhaj, Guy Carrault

**Affiliations:** 1 Laboratoire Traitement du Signal et de l’Image, Université de Rennes 1, F35000 Rennes, France; remo.lazazzera@gmail.com; 2 Institut National de la Santé et de la Recherche Médicale, U1099, F35000 Rennes, France; 3 Farasha Labs, 75000 Paris, France; yassir@thesowatch.com

**Keywords:** blood pressure, photoplethysmogram, pulse transit time, PPG, PTT

## Abstract

We present a novel smartwatch, CareUp${}^{®}$, for estimating the Blood Pressure (BP) in real time. It consists of two pulse oximeters: one placed on the back and one on the front of the device. Placing the index finger on the front oximeter starts the acquisition of two photoplethysmograms (PPG); the signals are then filtered and cross-correlated to obtain a Time Delay between them, called Pulse Transit Time (PTT). The Heart Rate (HR) (estimated from the finger PPG) and the PTT are then input in a linear model to give an estimation of the Systolic and Diastolic BP. The performance of the smartwatch in measuring BP have been validated in the Institut Coeur Paris Centre Turin (ICPC), using a sphygmomanometer, on 44 subjects. During the validation, the measures of the CareUp${}^{®}$ were compared to those of two oscillometry-based devices already available on the market: Thuasne${}^{®}$ and Magnien${}^{®}$. The results showed an accuracy comparable to the oscillometry-based devices and they almost agreed with the American Association for the Advancement of Medical Instrumentation standard for non-automated sphygmomanometers. The integration of the BP estimation algorithm in the smartwatch makes the CareUp${}^{®}$ an easy-to-use, wearable device for monitoring the BP in real time.

## 1. Introduction

A normal Systolic Blood Pressure (SBP) is below 120 mmHg and a normal Diastolic Blood Pressure (DBP) is lower than 80 mmHg. Raised Blood Pressure (BP) throughout its range is the most significant cause of death and disability in the world [1]. In the USA, about 30% of the population suffers from hypertension and less than 50% of them monitor their BP [2]. Increased pressure in the arteries is a common condition, leading to hypertensive heart disease and Cardiovascular Diseases (CVD). Accurate BP measurement is, therefore, vital in the prevention and treatment of such diseases, especially in hypertensive patients. Even if the BP is high, the patient probably will not have any symptoms: The first symptom of untreated high BP may be a heart attack, stroke or kidney damage. For this reason, it is often called the “silent killer”. In these circumstances, an easy-to-use home BP monitor is needed in many situations, especially for persons with stage 2 hypertension (140–90 mmHg), who need to keep track of their BP at home, which helps to find out if the treatment is working.

The most used method in medicine for BP estimation is to use a sphygmomanometer; then the hearing of the Korotkoff sounds (K-sound) allows a non-invasive BP measurement [3]. This method is not automatic and needs a person, such as as a doctor or a nurse, to perform the measurement.

Automatic devices still use a cuff wrapping around the arm and to give an estimate of the BP, they detect the pressure oscillations during cuff-deflation, using a built-in pressure sensor. For these devices, the Mean Arterial Pressure (MAP) is estimated using the amplitude variations of the oscillations and, from those, the SBP and DBP are deduced [4,5]. A recent study by Leung et al. indicated that over three in ten home BP monitoring cuffs were inaccurate [6]. Moreover, they cannot perform continuous measurements and they are difficult to integrate with wearable technologies.

In this paper, we used two Photoplethysmogram (PPG) signals as source for the BP estimation. These signals require a pre-processing step to filter and remove movements artifacts. The system proposed in [7] to filter PPG signal provides a reliable system for assessing the PPG physiological parameters and their monitoring over time for robust medical assessment. It is a bio-inspired nonlinear system, based on a reaction-diffusion mathematical model, implemented by means of the Cellular Neural Network (CNN) methodology. For the development of the device described in this work, embedding this system would require an important computational cost. For this reason, we decided to opt for a lighter and classical filtering method.

The study in [8] presents a very recent work on BP estimation based on PPG wearable sensors, that exploits the algorithm in [7]. In the study, a novel algorithmic approach is presented to estimate SBP and DPB: it is non-invasive, cuff-less and does not require user calibration. The method measures the BP through careful neural and mathematical analysis of the PPG signals. The PPG data are processed with an ad hoc bio-inspired mathematical model that estimates systolic and diastolic pressure values. In this case, the results showed about 97% accuracy. We tried to reproduce similar results but with a different mathematical model that could be embedded in wearable systems.

In [9] a BP estimation method, based on the physical model of wrist skin tissues and pulse wave velocity is proposed. These features enable long-term BP monitoring without incurring the limb compression caused by a cuff. Thus, this method is useful for individuals requiring continuous BP monitoring. The percentage errors of diastolic and systolic BP readings were, respectively, 4.74% and 4.49% at rest.

In a further study [10], the PPG signal was replaced by the Impedance Plethysmography (IPG) signal and was used to detect the Pulse Transit Time (PTT). The results showed that the change of the systolic pressure had a better relationship with the change of the PTTIPG than that of the PTTPPG (r = 0.700 vs. r = 0.450). Moreover, the IPG ring with spot electrodes would be more suitable to develop with the wearable cuffless BP monitor than the PPG sensor.

Another potential noninvasive cuffless method to estimate BP, is through Bioimpedance (BI) measurement. It reflects the change in BP through the change in the arterial cross-sectional area. The study in [11], proposes and examines a horizontal BI structure based on two sets of four-electrode BI interface, attached around the wrist. The measured PWVs correlate well with the BP standard device at 0.81 ± 0.08 and 0.84 ± 0.07 with low root-mean-squared-errors at 7.47 ± 2.15 mmHg and 5.17 ± 1.81 mmHg for SBP and DBP, respectively.

The study in [12], instead, investigated the Pressure Pulse Wave (PPW) signals collected from one piezoelectric-induced sensor located at a single site for cuffless BP estimation. Twenty-one features were extracted from PPW that collected from the radial artery and then a linear regression method was used to develop BP estimation models by using the extracted PPW features. The experimental results indicated that the mean ± standard deviation errors for the estimated SBP and DBP were 0.70 ± 7.78 mmHg and 0.83 ± 5.45 mmHg. The proposed model also demonstrated a high level of robustness in a maximum 60-day follow-up study. In terms of production costs, the PPG sensor is more convenient and easier to integrate in the electronics than the above mentioned technologies.

Here, we present a new, cuff-less method for BP estimation that exploits two PPG signals as input. It has been embedded in the CareUp${}^{®}$ smartwatch developed by Farasha Labs. This method is based on the Time Delay, it takes for the blood volume (Pulse) to travel from the heart to peripheral organs. This Time Delay is called PTT or Pulse Arrival Time (PAT) [13,14,15]. The PAT is obtained using an Electrocardiogram (ECG) recording device and a pulse oximeter (PPG) at a peripheral organ [16,17]. It can be shown that PAT is the sum of the Pre-Ejection Period (PEP) and PTT. The PEP is the time instants the electric signal needs to propagate and be converted into mechanical force, to squeeze the heart and open the aortic valve [18,19,20,21].

The PTT can also be estimated from two PPG: one acquired on a place proximal to the heart and another one on a distal place to it. In this case, the PTT is obtained recording the different time instants the pulse arrives at the two locations and then differentiating them. Using as input the heart rate (HR) estimated from one of the two PPG available and the PTT, it is then possible to estimate the BP. It is worth mentioning here, that the BP depends on different factors, such as vasomotor tones, neural control and HR. It is for this reason that the model we used also includes, as an additional term, the HR [22,23]. Moreover, this approach requires a specific calibration for each user and it seems that the model is dependent to temporal trials, as well as to motion activities [24,25]. Lastly, the accuracy measured by the regression coefficient ($R^{2}$) is low, with significant value variations even in the same subject at the same activity level [26,27], but a more-sophisticated model may lead to a loss in generalization and make the model subject-specific.

Different mathematical models have been proposed to model the Time Delay and BP [28,29,30]. Some of them will be presented, followed by the principles of measurements of the proposed method. The architecture of the device is then analyzed with the algorithm for the estimation of the BP. The software has been embedded in the CareUp${}^{®}$ smartwatch: the ease-of-use and portability of this device make it a perfect solution to check the BP anywhere the user needs. In the last part, we show the validation of the measurements performed on five control subjects at Farasha Labs and on forty-four patients in the Institut Coeur Paris Centre Turin (ICPC) in Paris. In the same clinical context of ICPC, the measures of the CareUp${}^{®}$ have been also compared to those of two commercial oscillometry-based devices: Thuasne${}^{®}$ and Magnien${}^{®}$. In the Appendix A is presented the user guide to calibrate the device and perform the BP measurement.

## 2. State of the Art

### 2.1. Principle of Measure

In this part, we are going to introduce the principle of measure, the estimation of the BP and the state of the art of the methods to estimate the BP using the PTT.

The electro-mechanical signal generated in the human heart by ventricular contraction produces a systemic wave of pressure into the arterial tree, called SBP. The pressure in the artery between two beats is the DBP. The pressure wave varies periodically between these two extremes and it causes dilation of the arterial walls. Moreover, on its path, it moves faster than the blood flow [18,31]. This pressure can be detected by measuring the variation of the oxygen content of the blood, caused by influx of oxygenated blood on the arrival of the pressure wave.

The concept of modern PPG measurement is originated from Aoyagi [32] and Yoshiya [33]. They associated the time variant PPG signal with arterial blood volume change and they assumed that venous blood did not pulsate. The PPG signal is used to determine and register the variations in blood flow in the body, which occur at each heartbeat. The PPG is captured by a pulse oximeter that is composed by a light source and a light detector: It detects the cardio-vascular pulse wave that propagates through the body.

The light source emits light at a certain fix wavelength and the amount of the back scattered light to the light detector corresponds with the variation of the blood volume. In this way, we are able to know the percentage of oxygen saturated hemoglobin with respect to the whole quantity of hemoglobin in the volume. This is because of the different absorption properties of the oxy- and deoxy-hemoglobin [34].

**Figure 1:**
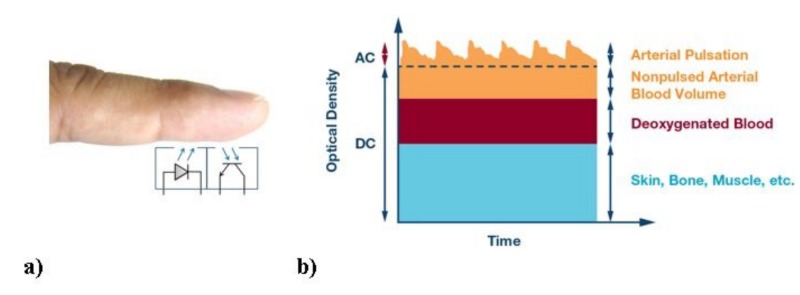
Principles of photoplethysmograms (PPG) measurement. (**a**) Schematics of the LED and the receiver: the LED emits light at the specific wavelength at which the absorption of the oxy-hemoglobin is maximum; the receiver collects the back scattered light [36]. (**b**) The PPG signal is composed by a DC component, due to the not-changing part in the tissue and the AC component due to the blood whose concentration of oxy- and deoxy–hemoglobin changes as the pulse [37].

The PPG signal has an AC and a DC component [33,35]. The AC component is the result of pulsating changes in arterial blood volume that is synchronous with the heartbeat. The DC component is related to the average blood volume and to the tissues. The AC component must be filtered out from the DC component in order to get the needed pulse signal. These concepts are visualized in Figure 1.

The arrival of the pressure wave is visible as the first peak on the PPG waveform [38]. The direct wave of the PPG waveform (systolic component) is the result of pressure transmission from the aortic root to the distal place where the signal is acquired. The second part (diastolic component) is formed by pressure transmitted from the ventricle along the aorta to the lower body where it is reflected back along the aorta to the distal place. The upper limb provides a common channel for both the directly transmitted pressure wave and the reflected wave and, therefore, has little influence on the contour of the PPG signal [34].

The central arteries push blood to narrow distal arteries by expanding during systole and contracting during diastole [39]. The elasticity of arteries determines the propagation speed: the Pressure Wave Velocity (PWV). The Bramwell–Hills and Moens–Kortweg’s equation, represents the relationship between pressure (P) and PWV; hence, the Time Delay (TimeDelay) for an artery with a length *L* [31]:

(1) $$PWV=\frac{L}{\text{TimeDelay}}=\sqrt{\frac{hE}{\rho d}}=\sqrt{\frac{hE_{0}e^{\alpha P}}{\rho d}}.$$

This equation indicates that the rise in pressure, with other parameters constant, will result in an increase in PWV and in an inverse effect on the Time Delay. The elasticity *E* of arteries determines the propagation speed. So, it is possible to express PWV in terms of fluid Pressure (*P*) by including blood density (ρ), artery diameter (*d*), artery thickness (*h*), α as a vessel parameter (Euler number) and $E_{0}$ the Young’s modulus for zero arterial pressure elasticity.

### 2.2. Estimation of the BP

In this context, the PTT refers to the time, taken by a pressure wave, to travel between two arterial sites and is inversely related to BP. It can be obtained by observing two distant PPG waves. Common sites used for these measurements are usually ears, toes and fingers [40].

The Bramwell–Hills and Moens–Kortweg’s equations give a logarithmic relationship between BP and the Time Delay. Assuming constant for a subject: the density of blood (ρ), the diameter of artery (*d*), the thickness of the artery (*h*), the distance at which the Time Delay is obtained (*L*) and the elasticity parameter ($E_{0}$), we can have the relationship of BP and the Time Delay represented as:

(2) $$BP=a*log_{e}\left(\text{TimeDelay}\right)+b.$$

We can differentiate Equation (2) with respect to time [16] to obtain:

(3) BP=a*(TimeDelay)+b.

Here, *a* and *b* are subject-specific constants and they can be obtained through a regression analysis between the reference BP and the corresponding Time Delay [41]. The mathematical relationship between BP and the Time Delay used in this work is a Linear Model that also exploits the HR as a second variable. Then, the model is:

(4) BP=a*(TimeDelay)+b*HR+c.

Several other studies have integrated the linear BP algorithm (Equation (3)) with other influencing factors, such as HR and Arterial Stiffness Index (ASI), that would affect the BP [17,19,42]. The effect of variance in HR has shown both positive and negative impacts on BP in clinical data. In normal conditions, it has a positive relation, but under baroreflex activity (the mechanism to regulate acute BP changes via controlling HR), HR is negatively correlated to BP [42,43,44].

In [41] the authors compared four different models, exploiting the PAT delay, for the BP estimation on the Boston’s Beth Israel Deaconess Medical Center (BIDMC) database. In particular, they used a logarithmic model, a linear model and two polynomial models. The average error and standard difference between the PAT-based method and the reference value was for DBP measurements 0 ± 2.13, while for the SBP, it was 1.3 ± 7.02. The larger disagreements between reference and measured SBP and DBP values for some of the data points was due to inclusion of data of hypertensive patients.

Another study using PAT and polynomial models has been presented in [45]. It is a subject-specific study with parameters that also depend on the height of the subject. The results showed an average error and standard deviation of 0.0790 ± 11.32 in the estimation of the SBP.

A work worth mentioning is presented in [46]. It studies a subject-specific approach with a more complex polynomial model in PAT. The results showed an error of 0.6 ± 9.8 for SBP and 0.9 ± 5.6 for DBP.

An exponential model has been, instead, used in the work [47,48] exploiting the PTT. In these cases, the results showed an error of −1.49 ± 6.51 for DBP and 2.16 ± 6.23 for SBP. The equation parameters were computed for a demographically similar group. This study was the closest to the approach proposed in this work and our results showed an improvement, even for an heterogeneous population.

The following part shows the methods deployed for the BP estimation and the architecture of the CareUp${}^{®}$ device. The signal processing technique is then analyzed and the linear model for the BP estimation is presented.

## 3. Methods

### 3.1. CareUp${}^{®}$ Architecture

**Figure 2:**
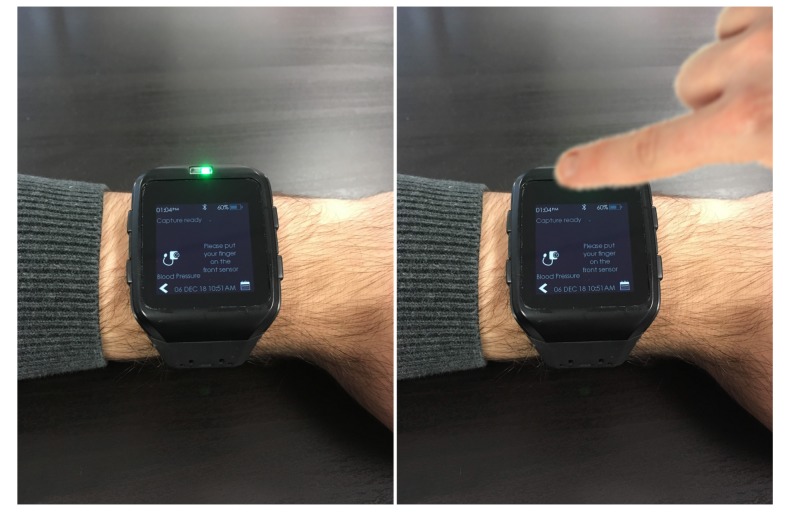
The CareUp${}^{®}$ smartwatch, where the algorithm for detecting the blood pressure (BP) has been embedded. One PPG waveform is taken from the back sensor of the watch in contact with the wrist skin and the second one is acquired by positioning the index finger of the other hand on the front oximeter sensor.

To understand the constraints of the smartwatch CareUp${}^{®}$, it is important to know some specifics of its hardware. It has a 32 bits MicroChip${}^{®}$ processor and two PPG sensors by Maxim Integrated™: one mounted on the back and one on the front of it. Figure 2 shows the CareUp${}^{®}$ smartwatch ready for the BP estimation task. The CareUp${}^{®}$ sensors use a green LED emitting at the wavelength of 537 nm and a photodetector with a radiant sensitive area of 1.38 mm${}^{2}$. The measures in this case were performed by wearing the watch on one arm and placing the index finger of the other hand, on the front sensor. The processing time was another constraint of the smartwatch, due to the fact that during the entire measurement the watch is only executing the BP task. The available RAM (32 KB) in the device was not enough to store the signals and perform the computation. For this reason, the signals were acquired by the two pulse oximeters for 30 s, filtered in real time and stored in the internal flash memory (512 KB). Only the correlation function was then allocated in the RAM to compute the Time Delay.

**Figure 3:**
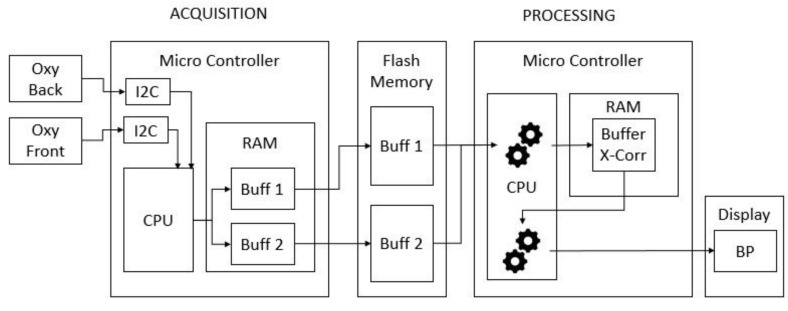
Hardware architecture of CareUp${}^{®}$ for BP estimation.

Figure 3 shows the hardware architecture for the whole process. We can describe the process as divided in two parts: the acquisition and the processing.

During the acquisitions, the drivers of the back and front oximeters give the values of the measure by I2C communication to the CPU. The CPU stores those values, first in two temporary buffers in the RAM: one for the back and one for the front sensor. The capacity of these two buffers is limited by the RAM size and cannot contain the whole data acquisition of 30 s. For this reason, when the buffers are full, they are empty in two bigger buffers in the flash memory.

The processing task begins when the acquisition process ends. This task uses the values stored in the external flash memory to perform the cross-correlation. This function is then stored in a buffer in the RAM.

In the following sections, we are going through the explanation of how we compute the Time Delay from the cross-correlation function, the HR and finally the BP, using the linear model.

### 3.2. Estimation of the BP

**Figure 4:**
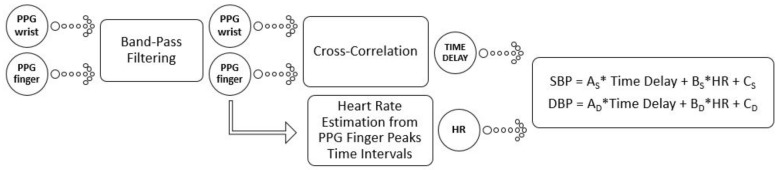
Diagram showing the BP estimation algorithm. SBP = systolic blood pressure; DBP = diastolic blood pressure; HR = heart rate.

The signal processing scheme is reported in Figure 4. After the filtering phase, two steps followed: one for identifying the Time Delay and another one to estimate the HR. The best technique to estimate the Time Delay revealed to be the cross-correlation between the two input signals. Nevertheless, we explored some other alternatives, such as those proposed by Laguna et al. in [49], to obtain the PPG Time Delay. Specifically, we also used the time difference between the points of maximum positive dependency and the time difference between the feet of the PPG waveforms. A comparison, on our Training Database, shows heavier computational costs in BP estimation by using these approaches. At this point, due to the lighter model criteria for portable technology, we assumed that there was no importance in the returns observed in the diastolic period and we imported only the aortic artery exit impulse observed in the systole period.

The last step of the schematic is, then, the estimation of SBP and DBP from a linear model, in the variables HR and Time Delay. All these steps are described in much more detail in the next sections and a user guide is presented in the Appendix A.

### 3.3. Filtering

The first processing step for the stored data was to filter the signals using a second order pass-band Butterworth filter with zero phase distortion, at the frequencies of 0.35 Hz and 6 Hz. The idea is to remove the DC component of the signal and all the frequencies out of the order of the pulse. The high frequencies, in fact, bring some dirt in the signal making it harder to recognize its features.

### 3.4. Time Delay Estimation

At this point, the next step was to detect the Time Delay between the two signals and for that the cross-correlation method of the two PPG signals was used.

The cross-correlation method allows us to establish at which time instance the two signals are more “similar”. The cross-correlation between two discrete signals f[m] and g[m] is computed as follows:

(5) $$\left(f☆g\right)\left[n\right]=\sum_{m=-inf}^{inf}\bar{f[m]}g\left[m+n\right],$$

where $\bar{f[m]}$ denotes the complex conjugate of f[m] and *n* is the displacement, also known as lag.

Once we obtained the cross-correlation function, the temporal position of its maximum tells us the time instant at which the similarity is the most. To ensure a good detection at all times, three consecutive peaks from the cross-correlation signal are selected. These three peaks should satisfy the following conditions:

- the first peak should correspond to a negative delay
- the second one to the maximum in amplitude peak
- the last third peak should be a positive delay peak

**Figure 5:**
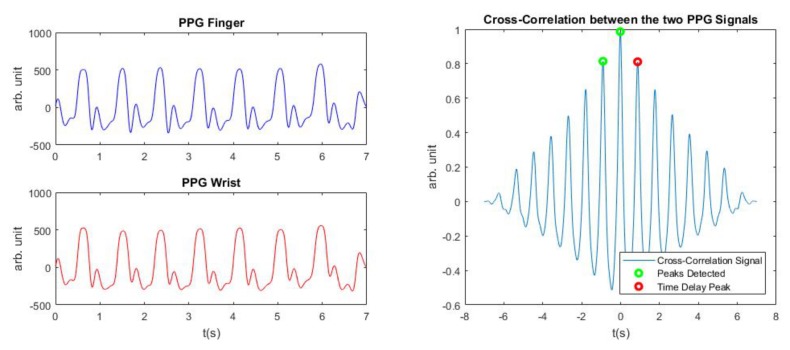
Two PPG signals: one from the wrist and one from the finger, used to perform the cross-correlation between the two signals.

If these conditions are not met at the first attempt, then we iterate the same procedure but choosing as second peak, the second biggest peak in the cross-correlation signal and so on. In the end, the Time Delay was always chosen as the time corresponding to the third peak. This criterion has been added in order to avoid a Time Delay measure less than zero and to have a number of the order of hundreds of milliseconds, as the models we adopted, expected. This procedure is visualized in Figure 5. Following this decision, if the second peak (of the three peaks we chose) is negative, the Time Delay corresponds exactly to the PTT:

(6) TimeDelay=PTT.

In the case the second peak is positive, then the Time Delay corresponds to a HR period plus the PTT:

(7) TimeDelay=HR+PTT.

Negative values of PTT are possible in this case. This mean that the pulse arrives first on the finger and then on the wrist. As explication, we formulated the hypothesis that what we capture during the recording on the wrist is not the arterial blood, but the venous return.

### 3.5. HR Estimation

To detect the HR, it was initially needed to extract the peaks of one of the two PPG waveforms. As preference, we used the PPG waveform of the finger because this signal was clearer due to the easier path for the light to penetrate the tissue. Once the peaks were extracted, the time differences between the peaks were computed obtaining a sequence of time differences. This sequence Δ*t* was then cleaned by forgetting the outliers that were not in the interval:

(8) Δt∈[m−3σ,m+3σ].

In this way, the atypical values of Δt were eliminated.

The HR was then estimated as the average of the time differences sequence. The result in Beats Per Minute (BPM) was evaluated as:

(9) BPM=Δt*60.

This value was then used as input to the linear model, for the estimation of SBP and DBP.

### 3.6. SBP and DBP Linear Model

The model has been built by linear regression analysis and its constants were evaluated by Manlises et al. [50]. The linear formulation of the model in the variables Time Delay (Delay) and HR is reported in the following equations:

(10) $$SBP=A_{S}*\text{Delay}+B_{S}*HR+C_{S},$$

(11) $$DBP=A_{D}*\text{Delay}+B_{D}*HR+C_{D}.$$

These equations cannot be universal and need to be subject-specific. Different subjects tend to have different physiological characteristics that reflect in different constants of Equations (10) and (11). The constants *A_S_* and *A_D_* were experimentally adjusted for each patient during the calibration procedure.

### 3.7. Calibration

For each new user, a new calibration is needed. The calibration aim is to center the measure around the Time Delay value of the subject and its BP. It is a one-time procedure that measure the PPG Time Delay ($Td_{\mathrm{cal}}$) and HR ($HR_{\mathrm{cal}}$). It also asks the user to input the SBP ($SBP_{\mathrm{cal}}$) and DBP ($DBP_{\mathrm{cal}}$), measured by a reference device in that moment. Then the algorithm receives these parameters and adds an offset to the constants $A_{s}$ and $A_{d}$: in this way, when the model receives as input $Td_{\mathrm{cal}}$ and $HR_{\mathrm{cal}}$, it gives the same values of SBP and DBP measured by the reference during the calibration. A practical user guide, to perform the calibration, has been added in the Appendix A.

## 4. Results

The algorithm has been embedded in the CareUp${}^{®}$ smartwatch and all the measures to validate the algorithm were directly performed with it. The whole BP estimation procedure lasts about 45 s. The watch starts storing the signal after 6 s from the beginning of the record. This is for cutting the beginning part of the signal and to wait for the filter to converge. Then we store 30 s of the filtered signal and the elaboration is performed in around 10 s.

### 4.1. Database

The validation was performed on two databases: the Learning Database (LDB) and the Test Database (TDB).

The LDB consisted of 22 acquisitions on five healthy male persons (mean age 34 ± 4 years) at Farasha Labs.

Each person was informed about the study and gave their oral consent before the experiment.

The measures were performed with a sphygmomanometer as reference and the CareUp${}^{®}$, Magnien${}^{®}$ and Thuasne${}^{®}$ devices were used as comparisons. Magnien${}^{®}$ and Thuasne${}^{®}$ are commercial devices, both fully automatic, worn at the level of the wrist and employ an oscillometric algorithm for BP estimation. For this database, all the acquisitions were performed in sitting position, at rest, with the arm at the level of the heart for the sphygmomanometer and the wrist at the level of the heart for the other devices. In this scenario, the BP was measured by a member of the laboratory as previously instructed.

The TDB, instead, consisted of 126 acquisitions on 44 subjects. This second part of the validation was performed in a clinical context, in the ICPC in Paris. The measures were performed on 52 mixed subjects: males and females with age of 67 ± 13 years, with European, Caucasian and African skin color; some of them were in healthy status, while others had cardio arrhythmia, coronary occlusion, or the implant of a pacemaker. A total 126 acquisitions were conducted, with a mean of three acquisitions per patient, in a time interval of around 90 min. The reference device was a sphygmomanometer mechanically inflated by means of a manual pump. The compared devices were still CareUp${}^{®}$, Magnien${}^{®}$ and Thuasne${}^{®}$. In particular, the last two devices implemented an oscillometric algorithm for BP estimation at the level of the wrist. In this scenario, all the acquisitions were performed by a professional. As in a clinical context, the measures were conducted with the arm at the level of the heart and sitting, at rest, between the hours of 10 a.m. and 4 p.m. In the time between the measures, the patients were in the waiting room of the clinic, without constrictions. For the three comparison devices, the only change in the measurement conditions was to have the wrist at the level of the heart. All three devices were worn on the same wrist. Finally, a total of 126 measures were conducted using the sphygmomanometer and CareUp${}^{®}$; of the 126 measures, 78 were also performed using Magnien${}^{®}$ and 76 also using Thuasne${}^{®}$.

The Clinic accepted to test and compare the CareUp device for research purposes. There was no conflict of interest for the clinic to test the device. In the clinic, the professionals performing the BP recordings were informed about the study and all the necessary instructions to use the smartwatch were provided. It is worth mentioning that the study was inserted in the clinical routine of BP monitoring of the patients and the protocol that has been followed is the same, the clinic uses daily. The clinic protocol has not been altered by the test of the CareUp device. Before each study recording, the professional informed the patients about the research and then asked for a voluntary participation. In case of positive participation, a consent form was signed by the patient and their personal information and diseases were recorded in the TDB with the measurements. The patients identity was anonymised by a numeric code and annotated in the consent forms. A copy of the consent was provided to the patients and the original was attached to the patient’s clinical files. The large participation to this study can be explained by the fact that the participants could benefit from more BP recordings, thus a better health monitoring. In this study, the dignity, rights, safety and well-being of all participants were taken into primary consideration.

**Figure 6:**
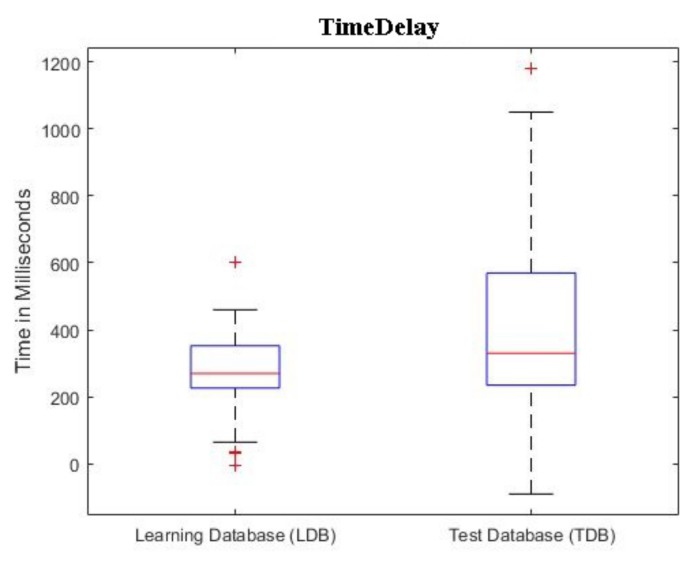
Time Delay boxplots for the Learning Database (LDB) and Test Database (TDB).

In Figure 6, we report the boxplots of the TimeDelay found for each patient, in both the databases. The computation of the TimeDelay follows the method depicted in Figure 5.

### 4.2. Estimation of SBP and DBP

The correlation factor using also HR in the linear model in Equation (4) has been found to be around 0.79 for SBP and 0.814 for DBP [42], confirming its significance. It is found that Chen’s algorithm, for a 4 min calibration interval, gives results according to the American Association for the Advancement of Medical Instrumentation (AAMI) standards and was unable to track large changes in BP, whereas Poon’s algorithm required shorter calibration intervals to maintain the same accuracy. The AAMI standard for non-automated sphygmomanometers allows a mean difference error of less than 5 mmHg and error standard deviation within 8 mmHg [51]. In our work, the need of a unique and fast calibration, led us to adopt the formulas found by Manlises et al. [50] for the model described in Equation (4):

(12) $$SBP=184.3-1.329*HR_{\mathrm{bpm}}+0.0848*Td,$$

(13) $$DBP=55.96-0.02912*HR_{\mathrm{bpm}}+0.02302*Td,$$

(14) $$Td=HR_{\mathrm{ms}}-\text{TimeDelay},$$

where $HR_{\mathrm{bpm}}$ is the HR in beats per minute and $HR_{\mathrm{ms}}$ is the HR in milliseconds. TimeDelay instead is the delay computed as in Equation (6) or (7). These two equations were chosen, among the others in [50], because they gave the best results for estimating the BP after the calibration.

During the Calibration procedure, the user input in the watch the SBP and DBP measured by a sphygmomanometer. We call this inputs $SBP_{\mathrm{cal}}$ and $DBP_{\mathrm{cal}}$. Then the watch computes the $HR_{\mathrm{ms}}$, $HR_{\mathrm{bpm}}$ (we can call it *HR*_bpm_cal_) and TimeDelay ($\text{TimeDelay}y_{\mathrm{cal}}$). So, using Equation (14), we obtain Td ($Td_{\mathrm{cal}}$).

By reversing Equation (12) and using SB_cal_ and HR_bpm_cal_, is possible to obtain the ideal Td ($Td_{\mathrm{SBPi}}$) for SBP. The same can be done by reversing Equation (13), introducing $DBP_{\mathrm{cal}}$ and HR_bpm_cal_: in this case we obtain $Td_{\mathrm{DBPi}}$. At this point we can define two constants:

(15) $$k_{\mathrm{SBP}}=Td_{\mathrm{SBPi}}\prime Td_{\mathrm{cal}},$$

(16) $$k_{\mathrm{DBP}}=Td_{\mathrm{DBPi}}-Td_{\mathrm{cal}}.$$

To calibrate the model, we add these offsets: $k_{\mathrm{SBP}}$ and $k_{\mathrm{DBP}}$, to TimeDelay in Equation (14), so the entire model becomes:

(17) $$SBP=184.3-1.329*HR_{\mathrm{bpm}}+0.0848*Td_{\mathrm{SBP}},$$

(18) $$Td_{\mathrm{SBP}}=HR_{\mathrm{ms}}-\text{TimeDelay}+k_{\mathrm{SBP}};$$

(19) $$DBP=55.96-0.02912*HR_{\mathrm{bpm}}+0.02302*Td_{\mathrm{DBP}},$$

(20) $$Td_{\mathrm{DBP}}=HR_{\mathrm{ms}}-\text{TimeDelay}+k_{\mathrm{DBP}}.$$

In this way, when the device computes a TimeDelay equal to $\text{TimeDelay}y_{\mathrm{cal}}$ and the $HR_{\mathrm{bpm}}$ corresponds to HR_bpm_cal_, the model outputs exactly $SBP_{\mathrm{cal}}$ in Equation (17) and $DBP_{\mathrm{cal}}$ in Equation (19). The offsets $k_{\mathrm{SBP}}$ and $k_{\mathrm{DBP}}$ are then stored in the memory of the device and added to every new TimeDelay computed by the smartwatch. This calibration method allows us to manage both the cases: when we acquire the signal on the arterial flow (Equation (6)) or on the venous return (Equation (7)).

The extraction of the features of the PPG waveform, as in [50], reveals it not to be an exploitable method in a portable device. This is because we cannot assure the user would pay attention for each measure and always take good quality signals. So, for the necessity of giving a ready result on each measure and to not discard any, we chose the cross-correlation method. In this case, the computational cost is heavier but well-performing. This led us to exclude other lighter methods to detect the Time Delay.

### 4.3. Criteria for Evaluation

A statistical analysis was done at first, by using the Bland–Altman plot to directly visualize the performance, then by calculating the mean and standard deviation of the errors and, finally, by performing the Wilcoxon rank sum test. In particular, the Wilcoxon test was performed only in the second part of the validation process, because the amount of data was more significant.

The Bland–Altman plot shows for each measure of BP, a point with coordinates:

(21) $$\left[x_{i}=\frac{i_{\text{reference}}+i_{\text{device}}}{2};y_{i}=i_{\text{reference}}-i_{\text{device}}\right]\left[\text{mmHg}\right].$$

The error of each measure *i* is then calculated as follows:

(22) $$e_{i}={\text{reference}}_{i}-{\text{device}}_{i}.$$

Then, applying the following formulas, we obtained the mean error $\bar{e}$ and standard deviation error $\sigma_{e}$ for each compared device, on *n* measures.

(23) $$\bar{e}=\frac{1}{n}\sum_{i=1}^{n}e_{i},$$

(24) $$\sigma_{e}=\sqrt{\frac{\sum_{i=1}^{n}{\left(e_{i}-\bar{e}\right)}^{2}}{n}}.$$

The results almost agreed with the AAMI standard for non-automated sphygmomanometers, which authorizes a mean error difference of less than 5 mmHg and a standard deviation error within 8 mmHg [51]:

(25) $$e_{i}\in 5\pm 8\left[\text{mmHg}\right].$$

Wilcoxon rank sum test, instead, tests if two independent samples come from identical continuous distributions with equal medians against the alternative that they do not.

### 4.4. Results on the LDB and TDB

Concerning the LDB, it can be said that the results are comparable to those of the devices already available in the market: Magnien${}^{®}$ and Thuasne${}^{®}$. The numerical values of mean error and standard deviation error are described in Table 1.

**Figure 7:**
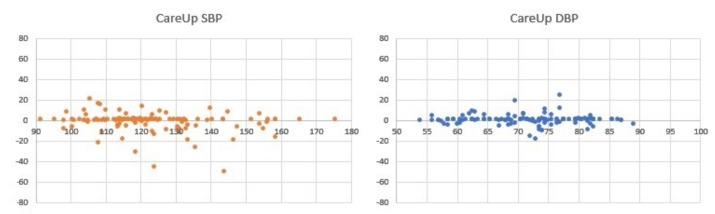
Bland–Altman plots showing the results comparison for SBP and DBP estimation on 126 measures from 44 subjects, taken in the Institut Coeur Paris Centre Turin (ICPC) in Paris. The acquisitions have been performed using a sphygmomanometer as reference device and the CareUp${}^{®}$ smartwatch.

**Figure 8:**
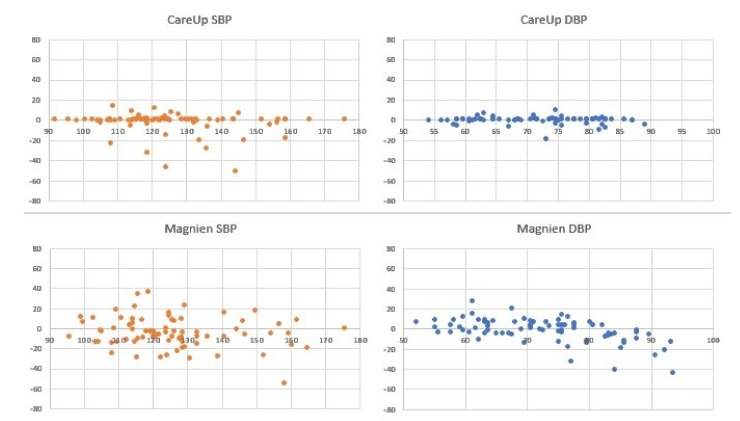
Bland–Altman plots showing the results comparison for SBP and DBP pressure estimation on 78 measures from 44 subjects, taken in the ICPC clinic in Paris. The acquisitions have been performed using a sphygmomanometer as reference device, Magnien${}^{®}$ and the CareUp${}^{®}$ smartwatch.

**Figure 9:**
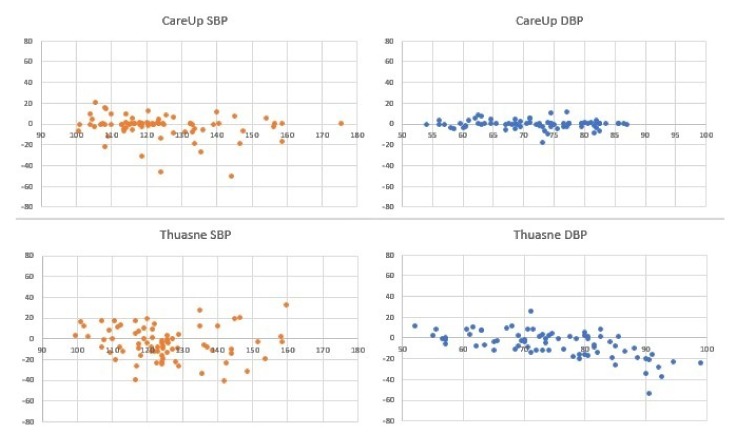
Bland–Altman plots showing the results comparison for SBP and DBP pressure estimation on 76 measures from 44 subjects, taken in the ICPC clinic in Paris. The acquisitions have been performed using a sphygmomanometer as reference device, Thuasne${}^{®}$ and the CareUp${}^{®}$ smartwatch.

For the results on the Test Database, Figure 7 and Table 2 report the comparison on 126 measures, between the sphygmomanometer and CareUp${}^{®}$. Figure 8 and Table 3, instead, report the results for the comparison, on 78 measures, among the reference, CareUp${}^{®}$ and Magnien${}^{®}$. Finally, Figure 9 and Table 4 report the comparison among the sphygmomanometer, CareUp${}^{®}$ and Thuasne${}^{®}$, on 76 measures.

This second part confirms the results obtained in the TDB and shows the robustness of the method respect to a diversity of physiological characteristics and pathologies. The Bland–Altman plots display some outliers, but the trend of the measures is around the value 0 of the y-axes. The results also put in evidence that there is no significant difference in the SBP measurement using market available devices and the CareUp${}^{®}$. Moreover, the DBP estimation performed using the smartwatch outperformed the other devices. The Wilcoxon test confirmed, by the *p*-values, that the measures taken with the reference and those taken with the CareUp${}^{®}$ smartwatch share the same median.

### 4.5. Statistical Tests

Table 2, Table 3 and Table 4 show the *p*-value of a two-sided Wilcoxon rank sum test. The null hypothesis is that the reference measures and those provided by the device are samples from the same identical continuous distribution and they share the same medians. In our study, the goal was to obtain a large *p*-value in order to not reject the null hypothesis. For example, if we obtain a *p*-value equal to 0.05, we can reject the null hypothesis with 95% confidence interval. In our tests, the *p*-values obtained with the measures of CareUp${}^{®}$ were always bigger than those obtained by the other two devices: Magnien${}^{®}$ and Thuasne${}^{®}$. For Thuasne${}^{®}$, in particular, we obtained *p*-values less than 0.05 and this was sufficient to reject the null hypothesis. We can conclude that the data distribution of the CareUp${}^{®}$ was the closest to the reference. In the end, as several experiments were conducted, these results show that the measurements of CareUp${}^{®}$ are reproducible.

## 5. Discussion

The CareUp${}^{®}$ is a smartwatch able to estimate the BP. The acquisition hardware is based on the use of two PPG sensors, whose output signals are used to estimate the PTT and HR. The PTT is defined as the time the pulse needs to reach two different distal parts of the body. The PTT and the HR are then used in a linear model to estimate the BP. For each user, a one-time calibration procedure is needed. It sets the BP estimation algorithm, according to the physiological parameters of the user.

The validation part was performed on five male subjects in Farasha Labs and on 44 subjects in the ICPC clinic in Paris. A sphygmomanometer was used as a reference and comparison devices used, were two commercial devices: Magnien${}^{®}$ and Thuasne${}^{®}$. A statistical analysis was conducted using the Bland–Altman, comparing the mean and standard deviation of the estimation errors and performing a Wilcoxon rank sum test. The AAMI for non-automated sphygmomanometers defines acceptable a mean error of less than 5 mmHg and a standard deviation within 8 mmHg [51] for SBP and DBP estimation. The average error on estimating the BP satisfies the AAMI standards. The standard deviation error, instead, agrees with the AAMI standards only for the DBP, while for the SBP reveals to be two points higher. With respect to the other two devices available in the market, CareUp${}^{®}$ shows the same performance in the estimation of SBP and even better for the DBP. Concerning the Wilcoxon tests, the CareUp${}^{®}$ outperformed the other devices in all the instances. This means that the measures performed with the reference device and those performed with the CareUp${}^{®}$ smartwatch share the same median and belong to the same distribution.

The results also show that there is no significant difference in the SBP measurement using market available devices and the CareUp${}^{®}$. This proves that the created design is accurate enough to be an alternative method in measuring BP. For ease-of-use and portability, CareUp${}^{®}$ is the optimal candidate for home BP monitoring. Further explorations can be done developing an oscillometric algorithm for the BP estimation (using the PTT variability) and comparing it with the proposed approach.

## 6. Conclusions

The validation of this BP estimation method was performed using the smartwatch in a clinical context. The results show that there is no significant difference in the SBP measurement using market available devices and the CareUp${}^{®}$. The clinical validation used in the study and reinforced by the Bland–Altman diagrams and the Wilcoxon test, shows that the developed device exceeds the two aforementioned commercial devices.

With the CareUp${}^{®}$ technology, it is possible to implement a PTT-based system that allows one to estimate their BP with the same accuracy of the market available devices, in a way that is easier to use and carry. The development of this kind of cuff-less BP monitoring is a novel solution in various medical scenarios.

Further, this system can be integrated in a continuous BP monitoring system, addressing healthy persons, as well as to CVD patients. In the same context, sleep apnea patients also require continuous BP monitoring and, thus, a wearable system with connectivity to the caregiver online network would be of great interest.

It is true that there are still many other wave features that can be extracted from the PPG signals. These are not addressed in this work because of the light computational costs required by the hardware. In spite of the above, this paradigm has already been solved today, thanks to the inclusion of interaction with cloud software, which allows more advanced processing with large volumes of data in real time, although it always requires an internet connection.

A weakness of these devices is that only records at rest were considered in the experimentation. This is due to the poor stability of the PPG signal to the movements.

The need of calibration represents a counter-factor and remains another weakness of the proposed approach. We can imagine that, at first, the calibration will be done at the medical office when the physician advises the patient to monitor their own BP. In the model, it is assumed that the HR does not change over time, considering a mean cardiac frequency for the model; however, in patients with hypertension, this parameter presents a high variability, which would require a constant calibration of the device. In a further step, we can collect a very large database of BP and Time Delay measurements in order to identify the most general models for the BP estimation. In this way, we can associate a specific model to each user, without the need for a calibration procedure. So, we suggest a collection of Time Delays and arterial pressures be included, so that the model has the capacity to interpret the changes in HR. Another unexplored area is represented by the development of an algorithm based on the PTT variability for BP estimation.

## Figures and Tables

**Table 1:** Mean error and standard deviation error for the twenty-two measures performed on five healthy male subjects in the Farasha Labs, with three different devices: CareUp${}^{®}$, Thuasne${}^{®}$ and Magnien${}^{®}$.

	Magnien${}^{®}$		Thuasne${}^{®}$		CareUp${}^{®}$	
	SBP	DBP	SBP	DBP	SBP	DBP
$\bar{e}$	−4.36	2	−8.36	−6.26	0.57	−1.31
$\sigma_{e}$	10.26	3	8.61	8.63	11.86	5.43

**Table 2:** Mean error, standard deviation error and *p*-value of data in Figure 7.

	CareUp${}^{®}$	
	SBP	DBP
$\bar{e}$	−1.52	0.39
$\sigma_{e}$	9.45	4.93
p	0.6338	0.7249

**Table 3:** Mean error, standard deviation error and *p*-value of data in Figure 8.

	Magnien${}^{®}$		CareUp${}^{®}$	
	SBP	DBP	SBP	DBP
$\bar{e}$	−3.44	−0.94	−1.97	0.31
$\sigma_{e}$	15.08	12.00	10.56	3.57
p	0.2328	0.8928	0.6952	0.7725

**Table 4:** Mean error, standard deviation error and *p*-value of data in Figure 9.

	Thuasne${}^{®}$		CareUp${}^{®}$	
	SBP	DBP	SBP	DBP
$\bar{e}$	−3.86	−5.42	−2.08	0.17
$\sigma_{e}$	15.34	16.16	11.67	4.45
p	0.0397	0.0296	0.6409	0.9222

**Figure A1:**
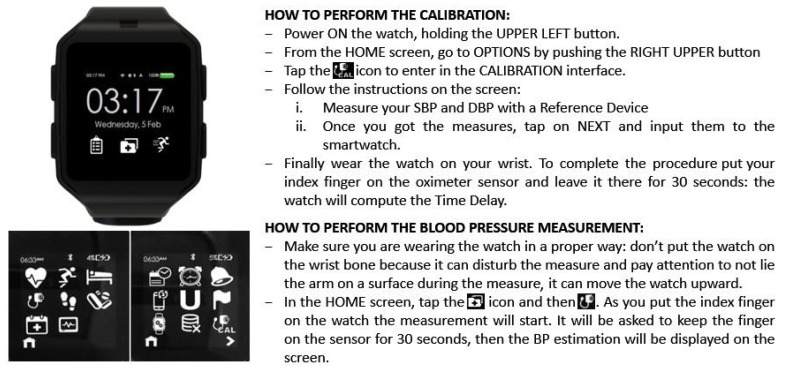
CareUp${}^{®}$ smartwatch home screen and watch panels for starting the BP measure and the calibration. In addition, the instruction manual to guide the user in the calibration and measurement procedure.

## Appendix A

Here, we describe a typical use case for the BP estimation via CareUp${}^{®}$. The instructions for the BP measure, as well as those for the calibration procedure, are described in Figure A1. For each new user a new calibration is needed and the watch can store only one calibration at a time. Thus, a new calibration will erase the previous one. The procedure is simple and we suggest to perform it periodically, as the body physiology changes over time due to different habits or medical treatments.
